# High Serum Lactate Dehydrogenase adds Prognostic Value to Cardiac Biomarker Staging System for Light Chain Amyloidosis

**DOI:** 10.7150/jca.30345

**Published:** 2019-09-07

**Authors:** Haiyan He, Jin Liu, Hua Jiang, Juan Du, Lu Li, Jing Lu, Weijun Fu

**Affiliations:** Myeloma and Lymphoma Center, Department of Hematology, Changzheng Hospital, Shanghai, China

**Keywords:** Light chain amyloidosis, Lactate dehydrogenase, Prognosis

## Abstract

**Objective**: To evaluate the impact of elevated serum lactate dehydrogenase (LDH) on prognosis of immunoglobulin light chain (AL) amyloidosis, and to investigate its prognostic value for the cardiac biomarker staging system.

**Patients and Methods**: We analyzed 83 consecutive patients with newly diagnosed immunoglobulin light chain amyloidosis who were treated with bortezomib or thalidomide based therapies between August 2010 and May 2017.

**Results:** Elevated serum LDH was identified to be associated with cardiac involvement, BNP and TNT (p=0.017, p=0.007, and p=0.026, respectively). The prognosis of patients with elevated serum LDH was inferior to that of patients with normal serum LDH. The two-year PFS rates of patients with elevated serum LDH and patients with normal serum LDH were 47.8% and 68.8% respectively (p=0.009), and the corresponding two-year OS rates were 51.5% and 73.9% respectively (p=0.007). We then incorporated serum LDH into the cardiac biomarker staging system involving cTNT and NT-proBNP. Patients were assigned a score of 1 of cTNT≥0.025ng/ml, NT-proBNP≥332ng/L, and LDH≥259U/L, creating a stage I to IV with scores 0 to 3 points, respectively. The proportion of patients with stage I, II, III, and IV were 31.6%, 32.9%, 21.1%, and 14.4%. The two-year PFS rates for patients in stage I, II, III and IV were 72.6%, 53.6%, 33.7% and 20%(p<0.001), respectively. The two-year OS rates of patients were 90.9%, 66.7%, 42.9%, and 20% (p<0.001), respectively.

**Conclusion**: Elevated LDH had adverse influence on prognosis of AL amyloidosis, which added prognostic value to the cardiac biomarker staging system.

## Introduction

Immunoglobulin light chain (AL) amyloidosis is one of plasma dyspraxia associated disease with deposition of immunoglobulin-derived amyloid in multiple organs 1,2. The outcome of patients with AL amyloidosis is highly dependent on the spectrum and severity of organ involvement, especially at cardiac level 3. Significant variability exists in the outcome among patients with similar clinical presentation. Soluble cardiac biomarkers including tropnin-T and NT-proBNP have been acknowledged as the best prognosis predictor of AL amyloidosis 4-6. In 2004 the cardiac biomarker staging system was firstly reported by Mayo clinic which including serum levels of cardiac troponins (troponin T [cTnT]) and N-terminal propeptide (NT-ProBNP) 7. The level of serum free light chain was also been found as an adverse prognostic factor of amyloidosis, which indicates that the risk stratification of patients depends not only on the burden of amyloid in the tissue (especially the heart) but also the size of plasma cells in bone marrow and its biology. To further identify risks of the patients, in 2012, Mayo clinic updated the staging system by incorporating serum free light chain into the cardiac biomarker staging system 8. However, there are still some other prognostic factors to be identified for further risk stratification of AL amyloidosis.

The prognostic value of lactate dehydrogenase is well understood in aseries of malignant neoplasms, especially in multiple myeloma and lymphoma. Elevated LDH (LDH ≥ upper limit of normal) is an important adverse indicator for survival of high-grade lymphoma and a parameter in the International Prognostic Lymphoma Index 9. In myeloma, elevated LDH is associated with lower response and shorter survival. It has been added to Revised International Staging System(R-ISS) of multiple myeloma as an important prognostic factor 10. In clinical practice, we observed a large portion of patients with AL amyloidosis also had an elevated LDH level. To evaluate the prognostic influence on AL amyloidosis and to investigate its prognostic value to the cardiac biomarker staging system, we retrospectively analyzed 83 patients, reported as below.

## Patients and Methods

### Patients

Between August 2010 and October 2016, a total of 83 patients with AL amyloidosis were investigated. Of these patients, 40 cases were associated with multiple myeloma. At least one biopsy specimen from endomyocardial tissue, kidney, rectum, bone marrow or subcutaneous fat was positive of amyloid, and the presence of amyloid was visualized by Congo red staining, producing apple-green birefringence under polarized light. Organ systemic involvement was defined by laboratory and clinical manifestations of renal, cardiac, hepatic gastrointestinal, neuropathic, pulmonary, or soft tissue involvement according to 2005 consensus criteria 11. All patients were provided written informed consent for this study. Approval was obtained by the ethics committee of the Shanghai Changzheng Hospital.

### Laboratory methods

Serum lactate dehydrogenase activity was determined by using Ectachem clinical chemistry slides with a multipoint rate slides test (Johnson &Johnson Clinical Diagnositic, USA) on Ectachem 259 analyzer.

Assays for cTnT was performed by sensitive second- and third-generation assays with reagents provided by Roche Diagnostics (Indianapolis, IN) and DADE (Newark, DE). The troponin T assay has a limit of detection of less than 0.01 g/L and coefficients of variability of 10% at 0.035 g/L and 20% at 0.015 g/L (Roche Diagnostics). The value of 0.035 g/L is the lowest value that meets the European Society of Cardiology/American College of Cardiology (ESC/ACC) criteria for precision. NT-proBNP levels were measured with electrochemilumi-nescence sandwich immunoassay (ECLIA; Roche) on an Elecsys System 2010.

### The Cardiac Biomarker Staging System

For patients with unavailable results of serum free light chain, the cardiac biomarker staging system involving NT-proBNP and troponin T levels was used. In this system threshold values were chosen (cTnT0.035 g/L; and NT-proBNP332 ng/L). Patients are considered stage I (low risk) when both troponin and NT-proBNP are below the threshold, stage II (intermediate risk) if only one marker is below the threshold, and stage III (high risk) if both are no less than the threshold.

### Treatment

Among the 83 newly diagnosed patients, 68 patients were treated with bortezomib-based regimen as induction therapy, and 15 patients were treated with thalidomide-based regimen as induction therapy.

### Statistical analysis

SPSS (IBM, version 16.0, New York, NY, USA) was employed to perform all analysis. Comparison among groups and comparison between two groups were performed by one-way ANOVA and chi-square test respectively. Survival analysis was performed using Kaplan-meier method. Differences were considered significant when the p-value was less than 0.05. OS was defined as the time from diagnosis to death for any cause, and PFS was defined as the time from the initiation of therapy to disease progression, relapse, and death for any cause.

## Results

### Patient characteristics

Baseline clinical characteristics of the 83 patients were shown in Table 1. The median age of the patients was 61 (range, 36-82), and 62 (74.7%) patients were male. 43 (51.8%) patients were primary AL amyloidosis and the other 40 (48.2%) patients were with multiple myeloma. 57 (68.7%) patients had cardiac involvement, 65 (78.3%) patients had renal involvement with levels of proteinuria over or equivalent to 500 mg, and 16 (19.2%) patients had renal insufficiency with elevated levels of serum creatinine over or equivalent to 176mmol/L. According to the Mayo cardiac biomarker staging system (2004), all patients had the two available variables with 33 patients in stage I, 31 patients in stage II, and 19 patients in stage III, respectively. FISH abnormalities were detected in 66 patients. The incidence rates of t(11;14), t(4;14), 17p-, 13q-, 1q21+ were 15.2%, 12.1%, 4.5%, 30.3%, and 43.9%, respectively.

The correlation between serum LDH and other clinical and laboratory parameters was investigated. Serum LDH was associated with the level of serum tropnin-T and NT-proBNP. The median value of c-TNT in patients with elevated level of serum LDH was 0.18(0-1.55) ng/ml, which was significantly higher than that of 0.01(0-0.048) ng/ml in patients with normal level serum LDH (p=0.026). The median value of NT-proBNP in patients with elevated level of serum LDH was 4650 (60.3-33400) pg/ml, which was significantly higher than that of 844 (48.4-9680) pg/ml in patients with normal level serum LDH (p=0.007). Accordingly the Mayo 2004 staging system incorporating NT-proBNP and troponin levels was also found to be correlated with serum LDH. 34.2% (13/38) patients with elevated level serum LDH were in stage III, which was significantly higher than that of 15.6% (7/45) in patients with normal level LDH (p=0.004). Cardiac involvement, defined by the level of NT-proBNP and echocardiography, was also found to be associated with serum LDH. 81.6% (31/38) patients with high serum LDH had cardiac involvement, which was significantly higher than that of 57.8% (26/45) in patients with normal level serum LDH (p=0.005). Other clinical parameters such as age, gender, hemoglobin, β2-MG, renal involvement and FISH results, have not found to be correlated with serum LDH.

### Survival and prognosis

**Figure 1:**
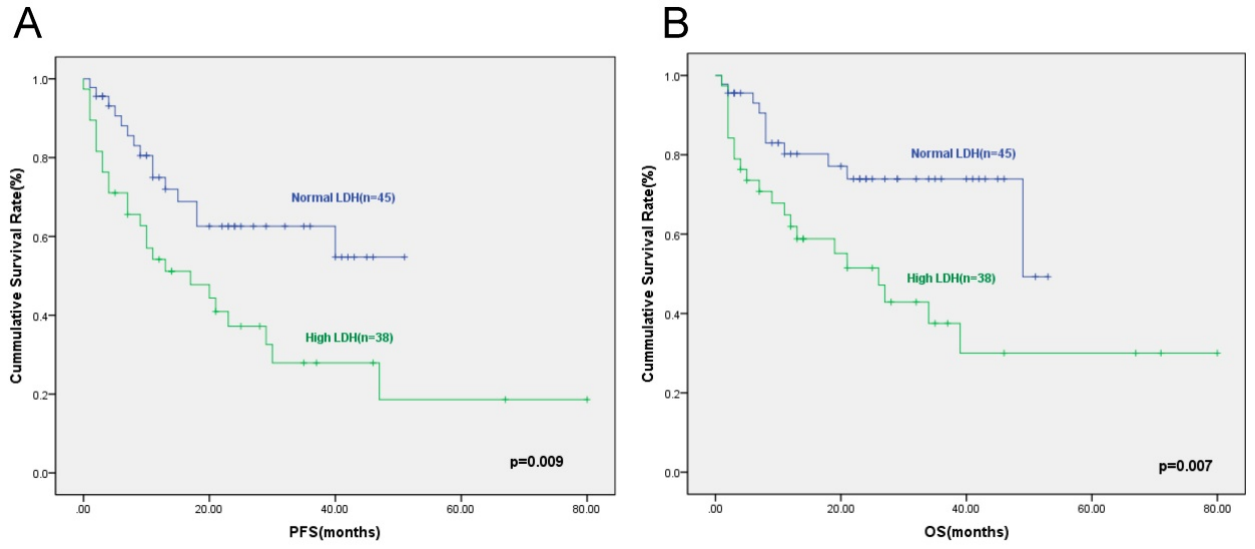
The progression free survival (A) and overall survival (B) according to normal or high serum LDH level. The two-year PFS rates for patients with normal serum LDH and high-level serum LDH were 68.8% and 47.8% (p=0.009), and the two-year OS rates for patients with normal serum LDH and high-level serum LDH were 73.9% vs 51.5%(p=0.007).

At a median follow-up of 16 (range,3-80) months, 32 (38.6%) patients died, 11 from the normal serum LDH group and 21 from the high-level serum LDH group, and the relative risk was 2.26. The two-year progression free survival (PFS) rates for patients with normal serum LDH and high-level serum LDH were 68.8% and 47.8% respectively (p=0.009) (Figure 1A), and the two-year overall survival (OS) rates for patients with normal serum LDH and high-level serum LDH were 73.9% and 51.5%, respectively (p=0.007) (Figure 1B).

**Figure 2:**
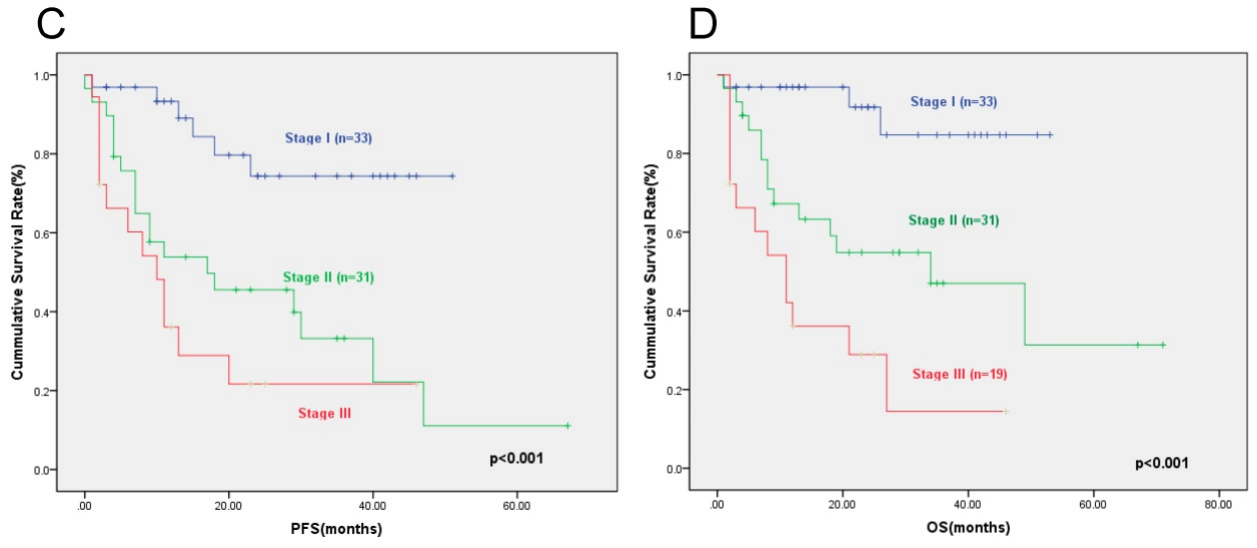
The progression free survival (C) and overall survival (D) according to the cardiac biomarker staging system. The two-year PFS rates for patients in stage I, II, and III were 74.4%, 45.6%, and 21.7% respectively (P<0.001), and the two-year OS rates were 84.7%, 47.0% , and 28.9% respectively (P<0.001).

According to the Mayo cardiac biomarker staging system (2004), all patients had available NT-proBNP and cTNT results with 33 patients in stage I, 31 patients in stage II, and 19 patients in stage III, respectively. The two-year PFS rates for patients in stage I, stage II, and stage III were 74.4%, 45.6% and 21.7% respectively (p<0.001) (Figure 2C). The two-year OS rates for patients in stage I, stage II, and stage III were 84.7%, 47.0% and 28.9% respectively (p<0.001) (Figure 2D).

**Figure 3:**
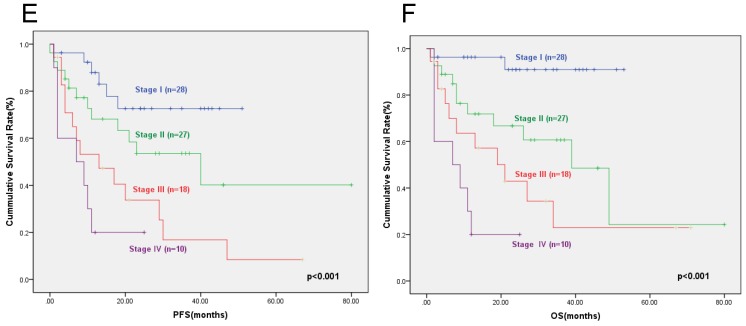
The progression free survival (E) and overall survival (F) according to the new staging system (incorporating serum LDH to the cardiac biomarker staging system).The two-year PFS rates for patients in stage I, II, III, and IV were 72.6%, 53.6%,33.7%, and 20% (p<0.001) (Figure 3E), and the two-year OS rates were 90.9%, 66.7%, 42.9%, and 20% (p<0.001) (Figure 3F).

To identify the patients with the worst prognosis, we incorporated serum LDH into the cardiac biomarker staging system. Patients were assigned a score of 1 of cTNT≥0.025ng/ml, NT-proBNP332≥ng/L, and LDH≥259U/L, creating a stage I to IV with scores 0 to 3 points, respectively. The number of patients in stage I, II, III, and IV were 28, 27, 18, and 10. and the two-year PFS rates for patients in stage I, II, III, and IV were 72.6%, 53.6%,33.7%, and 20% respectively (p<0.001) (Figure 3E). The two-year OS rates for patients in stage I, II, III, and IV were 90.9%, 66.7%, 42.9%, and 20% respectively (p<0.001) (Figure 3F).

## Discussion

AL amyloidosis is characterized by a relatively low burden of clonal plasma cells and involvement of multiple organs by immunoglobulin light chain-derived fibrils. The outcome of patients with AL amyloidosis is heterogeneous, and the median OS from diagnosis among all patients is approximately 1 year 12. Outcome of the disease is related to a series of factors. Cardiac involvement is the most significantly adverse predictor of survival. Hence, the mayo staging system (2004) for light chain amyloidosis adopted the of use serum levels of cardiac troponins (that is, troponin T[cTnT] or I) as well as brain natriuretic peptide (BNP) and its N-terminal propeptide 7. According to this staging system, the median survivals of patients in stages I, II, and III, were 26.4, 10.5, and 3.5 months, respectively. To assess the prognosis of AL amyloidosis more accurately and potentially select the optimal therapy, a new staging system incorporating both cardiac biomarkers and serum FLC measurements for AL amyloidosis was developed in 2012 8. The median OS of patients with stages I, II, III and IV disease from diagnosis was 94.1, 40.3, 14.0, and 5.8 months, respectively. Compared with the previous staging system, the revised mayo staging system was more developed by adding the level of clonal plasma cells because the underlying abnormality in AL amyloidosis is the clonal plasma cell. However, there were still prognostic factors needed to be identified to further stratify the patients.

In this study, we found that serum LDH level was also an adverse prognostic factor of AL amyloidosis. It is known that lactate dehydrogenase is a glycolytic enzyme which exists in all normal tissue cells as well as in tumor cells. Elevated serum LDH is a marker for tissue injury, inflammation, hemolysis and myocardial infarction 13-15, whose prognostic value has been shown in several malignancies such as germ cell tumors, lymphoma, melanoma and renal cell carcinoma 16-19. In this study, we found a total of 45.8% patients with AL amyloidosis had elevated serum LDH level. It needs to be further cleared whether the elevated serum LDH level indicted the degree of organ involvement or the number of clonal plasma cells. A significant correlation of elevated LDH with cardiac biomarker was also identified, which indicated the addition of LDH as a marker of tissue injury in AL amyloidosis. It is known that there are five types of LDH Isoenzymes, LDH1, LDH2, LDH3, LDH4, and LDH5 respectively. The serum LDH1 and LDH2 would be significantly upregulated in case of myocardial injury. Next, we should detect LDH Isoenzymes and check the relation of each of Isoenzymes with the prognosis of AL amyloidosis. Palladini et al. have demonstrated in a small set of patients that reductions in NT-proBNP occur in majority of patients who enjoy a hematologic response 20. As serum LDH is also a valuable predictor of overall survival in patients, serum LDH could be a potential probable response evaluating parameter in AL amyloidosis.

In this study we developed a novel framework by incorporating the serum LDH to the cardiac biomarker staging system. With this framework the highest risk patients are clearly separated from the lower risk patients. The highest risk patients with three high-risk factors (cTNT≥0.025ng/ml, NT-proBNP332≥ng/L, and LDH≥259U/L) had one-year survival rate of 18%. The separation of stage II and stage III is less dramatic than that in the Mayo cardiac staging system (2004). Using this system, we found a good separation between I, II, III and IV stage. Our study is limited to a relative small number of patients (83 cases) and short follow-up (median 16 months). Validations in other datasets with larger numbers of patients and longer follow-up would be sufficient for the poor prognosis of LDH in AL amyloidosis.

## Figures and Tables

**Table 1:** The correlation between serum LDH and other clinical characteristics.

	All patients (n=83)	Patients with High serum LDH (n=38)	Patients with Normal serum LDH (n=45)	P value
Age (median, range), years	61,36-82	59,42-76	61,36-82	0.506
Gender				
Male,%(n)	74.7(62)	65.8(25)	82.2(37)	0.072
Involved organ,%(n)				
Cardiac	68.7(57)	81.6(31)	57.8(26)	0.017
kidney	78.3(65)	76.3(29)	80(36)	0.443
Primary,%(n)	51.8(43)	(18/38)	(25/45)	
AL with MM, %(n)	48.2(40)	(20/38)	(20/45)	0.513
Renal disfunction, %(n)	19.3(16)	26.3(10)	13.3(6)	0.168
HGB, median (range) g/L	112(51-163)	113(70-163)	110(51-150)	0.410
BNP, median (range) pg/ml	1558 (48.4-33400)	4650 (60.3-33400)	844 (48.4-9680)	0.007
c-TNT (median, range), ng/ml	0.02(0-1.55)	0.18(0-1.55)	0.01(0-0.48)	0.026
Beta2-MG (median, range)	3.91(1.13-19.24)	5.1(1.54-19.24)	3.24(1.13-16.52)	0.061
Mayo stage (2004), % (n)				
I	39.8(33)	21.1(8)	55.6(25)	
II	37.3(31)	44.7(17)	28.8(13)	
III	2.8(19)	34.2(13)	15.6(7)	0.004
FISH				
t(11;14)	15.2(10/66)	14.3(5/35)	16.1(5/31)	0.501
t(4;14)	12.1(8/66)	11.4(4/35)	12.9(4/31)	0.530
17p-	4.5(3/66)	2.9(1/35)	6.5(2/31)	0.902
13q-	30.3(20/66)	25.7(9/35)	35.5(11/31)	0.276
1q21+	43.9(29/66)	51.4(18/35)	35.5(11/31)	0.146
